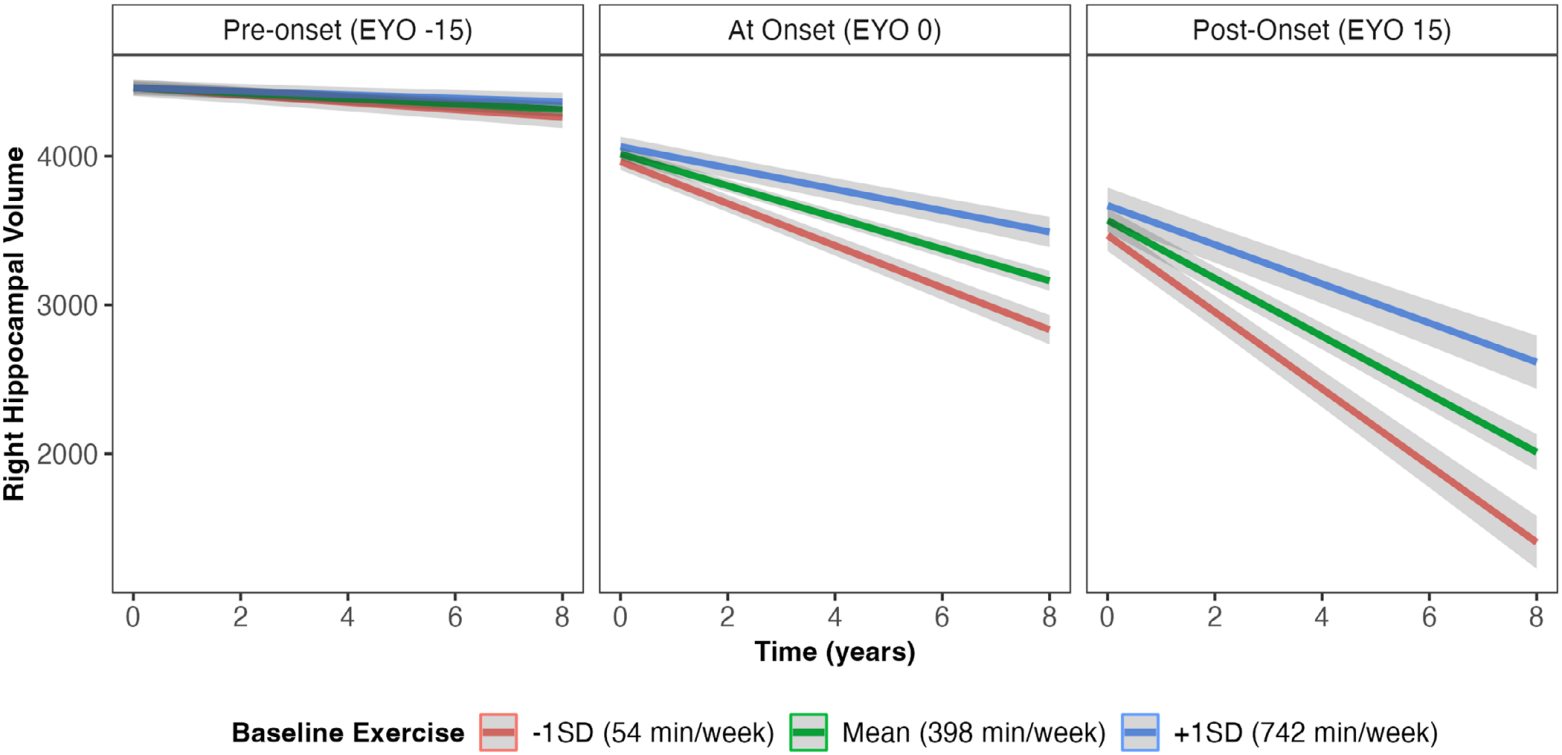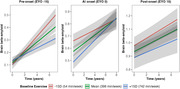# Longitudinal associations between self‐reported exercise levels and Alzheimer’s related biomarkers in Autosomal Dominant Alzheimer’s Disease

**DOI:** 10.1002/alz.085308

**Published:** 2025-01-09

**Authors:** Kelsey R Sewell, James D. Doecke, Hamid R. Sohrabi, Stephanie R Rainey‐Smith, Jeremiah J Peiffer, Randall J. Bateman, John C. Morris, Eric McDade, Ralph N Martins, Belinda M. Brown, The DIAN Study Group

**Affiliations:** ^1^ Murdoch University, Perth, Western Australia Australia; ^2^ The Australian e‐Health Research Centre, CSIRO, Brisbane, QLD Australia; ^3^ Department of Biomedical Sciences, Macquarie University, Sydney, NSW Australia; ^4^ Centre for Healthy Ageing, Murdoch University, Murdoch, Perth, Western Australia Australia; ^5^ Edith Cowan University, Perth, Western Australia Australia; ^6^ Alzheimer’s Research Australia, Perth, Western Australia Australia; ^7^ Australian Alzheimer’s Research Foundation, Perth Australia; ^8^ Centre for Healthy Ageing, Murdoch University, Murdoch, Western Australia Australia; ^9^ Murdoch University / Centre For Healthy Ageing, Perth Australia; ^10^ Washington University School of Medicine, St. Louis, MO USA; ^11^ Washington University in St. Louis, School of Medicine, St. Louis, MO USA

## Abstract

**Background:**

Accumulating evidence indicates exercise may delay or prevent the onset of Alzheimer’s disease (AD). To our knowledge, no study has investigated the longitudinal impact of exercise on AD‐related biomarkers in individuals with Autosomal dominant Alzheimer’s disease (ADAD) mutations who are destined to develop AD. This study examined longitudinal associations between self‐reported exercise levels and AD‐related biomarkers in a cohort of ADAD mutation carriers and investigated whether observed associations depended upon disease stage.

**Method:**

This prospective cohort study included participants from the Dominantly Inherited Alzheimer’s Network (DIAN). Participants were *n* = 308 ADAD mutation carriers aged 39.7 ± 10.8 years (56.5% female) with data available for self‐reported exercise participation and either brain imaging (hippocampal volume, total volume, gray matter volume, white matter hyperintensities, brain Aβ), or biomarkers quantified from cerebrospinal fluid (Aβ40, Aβ42, p‐tau, t‐tau, p‐tau/Aβ42, and Aβ42/40). Associations between exercise and AD biomarkers (i.e., from brain imaging and CSF) were examined using linear mixed models.

**Result:**

Greater weekly baseline exercise was associated with slower accumulation of brain Aβ at preclinical disease stages (β = ‐0.16, 95% CI ‐0.23 – ‐0.08), and slower decline of right and left hippocampal volume (β = 0.06, 95% CI 0.03–0.08; β = 0.06, 95% CI 0.02–0.09, respectively), total cortical volume (β = 0.03, 95% CI 0.01–0.05), total subcortical gray matter volume (β = 0.03, 95% CI 0.01–0.05), and total gray matter volume (β = 0.03, 95% CI 0.01–0.05), effects which became significant approximately 5 years before predicted cognitive symptom onset.

**Conclusion:**

These findings demonstrate that exercise is associated with more favourable profiles of AD‐related biomarkers in those with ADAD mutations. The causal direction of this research is difficult to ascertain, thus future study designs investigating the therapeutic potential of exercise in both ADAD and late‐onset AD should be considered before clinical recommendation of exercise is implemented.